# Role of archaea in human disease

**DOI:** 10.3389/fcimb.2013.00042

**Published:** 2013-08-13

**Authors:** Rustam I Aminov

**Affiliations:** Department of Basic Medical Sciences, Faculty of Medical Sciences, University of the West Indies at MonaKingston, Jamaica

A decade ago a hypothesis was proposed that, similar to the Bacteria and Eukarya, the archaeal domain of life might harbor certain species capable of causing disease (Cavicchioli et al., 2003; Eckburg et al., 2003). Now, a decade later, it is time to revisit the topic in the light of new data available. The last decade witnessed a massive use of molecular ecology tools in clinical microbiology, and these data can be inspected for the potential involvement of the Archaea in various infectious diseases in humans and animals.

Unlike the Bacteria, the diversity of the Archaea in the human body is substantially lower, including representatives of only one phylum, Euryarchaeota. The phylum includes three species: *Methanobrevibacter smithii*, found mostly in the gut and vagina (Miller and Wolin, 1982; Belay et al., 1990); *Methanosphaera stadtmanae*, found mostly in the gut (Miller and Wolin, 1985); and *M. oralis*, found mostly in the oral cavity (Belay et al., 1988; Ferrari et al., 1994). Compared to bacteria, the relative abundance of archaea is also much lower (Miller and Wolin, 1982), although the recent improvements in cultivation and DNA detection may help to estimate these numbers more accurately (Dridi et al., 2009; Khelaifia et al., 2013).

The first work suggesting an association of the Archaea with human gastrointestinal disease was published in 1985 (McKay et al., 1985). Patients with Crohn's disease, ulcerative colitis and primary pneumatosis intestinalis displayed a significantly lower incidence of methane excretion compared to healthy subjects. On the contrary, patients with diverticulosis showed a significantly increased incidence of methanogens compared to control (Weaver et al., 1986). Since then, there have been a number of studies, with more recent ones using molecular ecology markers, such as the 16S rRNA and *mcrA* genes, which have confirmed these two initial observations. That is, in conditions characterized by extended transit time in the intestine, the incidence, and rate of methane production are higher (Fiedorek et al., 1990; Pimentel et al., 2003; Chatterjee et al., 2007), while the diarrheal conditions of human gastrointestinal disease result in the opposite trend with lower incidences of methanogenic archaea and lower rates of methane production (McKay et al., 1985; Scanlan et al., 2008). Chemotherapy-induced diarrhea in cancer patients has also resulted in the decrease of methanogenic archaea in parallel with the loss of beneficial bacteria (Stringer et al., 2013). Indeed, experimental interventions to increase the intestinal transit rate have resulted in the decline of fecal methanogens and methane production, while the opposite effect has been observed in the loperamide treatment group (Lewis and Cochrane, 2007).

Recent reassessments of the role of methane production among patients with gastrointestinal disturbances have clearly associated elevated methane production with alterations in intestinal motility, such as constipation, but not with other conditions (Attaluri et al., 2010; Furnari et al., 2012). Thus, a pathogenic link with methanogens is unlikely (Di Stefano and Corazza, 2010), and their involvement in gastrointestinal disease is presently uncorroborated. From the ecological/system biology viewpoint the numbers and the methane production by archaea is more likely a reflection of syntrophic relationships in the gut where the local environment, depending on a number of factors, may favor hydrogen channeling through alternative mechanisms of hydrogen disposal, such as methanogenesis, sulfate reduction, or acetogenesis. In this regard, alternative generation of highly toxic hydrogen sulfide as a result of sulfate reduction in the gut may impose much higher health risks (Medani et al., 2011; Carbonero et al., 2012) compared to more inert methane.

The best-studied cases of a potential involvement of the Archaea in human pathology, however, are linked to periodontal disease. *Methanobrevibacter oralis*-like phylotypes, for example, have been detected by PCR in up to 36% of periodontitis patients (Lepp et al., 2004). In another study, five cases of apical periodontitis out of 20 total have been found positive for the Archaea (Vianna et al., 2006). The follow up work by the same group has greatly contributed to our understanding of the role played by hydrogenotrophic microorganisms in periodontal disease (Vianna et al., 2008). Compared to periodontitis patients, the supragingival plaque of healthy subjects harbors a lower total microbial load, and the hydrogenotrophic group is represented exclusively by acetogenic bacteria, also at lower numbers. On the contrary, the subgingival plaque from periodontitis patients harbors a larger number of total bacteria, and the hydrogenotrophic group includes methanogenic archaea and sulphate-reducing bacteria (SRB) in addition to acetogenic bacteria. The latter two groups are absent in healthy control subjects but present in 65% of periodontitis patients, alone or in combination (Vianna et al., 2008).

It needs to be noted here that the presence of SRB in human saliva can be detected in 30% of subjects and, among other oral and systemic conditions, the only statistically significant association of the SRB carriage is with periodontitis (Heggendorn et al., 2013). Although the proportion of hydrogenotrophs in periodontal disease is below 1% of the total microbiota (Vianna et al., 2008), the hydrogen sink they provide may create a favorable microenvironment for the microbial consortium, especially for the proliferation of a keystone periodontal pathogen, *Porphyromonas gingivalis*, which is capable of inducing host responses that ultimately result in uncontrolled inflammation and tissue damage (Hajishengallis et al., 2012).

Thus, the role of the Archaea in periodontal disease cannot be construed within the frames of a typical host-pathogen interaction, and we have to acknowledge that these are not *bona fide* pathogens. Their involvement in disease can still be interpreted from the point of view of polymicrobial diseases that has recently gained considerable attention (Peters et al., 2012). The diseases involve complex microbial communities instead of clear-cut cases of monocultural infections by classical pathogens.

The fine-tuned host-microbe interaction that has been evolving during the long co-evolutionary adaptations of both sides to one another is largely mutual but can be compromised due to genetic defects and/or environmental factors (Khachatryan et al., 2008; Littman and Pamer, 2011). Because of a malfunctioning interface, a subset of normally symbiotic bacteria could display potentially pathogenic properties; they thus have been called “pathobionts” to be differentiated from the “classical,” acquired or opportunistic pathogens (Chow et al., 2011). Can then the commensal methanogenic Archaea be considered as “pathobionts”? Pathogenic potential of pathobionts is expressed under certain circumstances, such as in a genetically susceptible host (Chow et al., 2011), and recent analyses have indeed found a significant association between IL-10 polymorphisms and periodontitis (Albuquerque et al., 2012; Atanasovska-Stojanovska et al., 2012; Zhong et al., 2012). A broader genome-wide association study, however, failed to detect any significant association of genetic polymorphisms with periodontitis diagnosis (Divaris et al., 2012). Suggestive evidence of association has been obtained for 13 loci and 8 periodontal pathogens from the previously defined “red” and “orange” clusters (Socransky et al., 2004). As for the methanogen carriage, a study of monozygotic and dizygotic twins found no link between the host genetics and the occurrence of methanogenic microbiota (Florin et al., 2000).

In a “classical” pathogen situation a taxonomic signature of it is sufficient to deduce its identity and associated etiology of a disease. In polymicrobial diseases, such as periodontitis, however, taxonomic signatures are less efficacious as disease predictors, although some attempts are being made to identify the key players within certain pathobiota (Hajishengallis et al., 2012). Still, the keystone pathogens rely on the community to realize the full pathogenic potential, and the hydrogen sink appears to be important for the periodontal disease progression. The role of hydrogenotrophic microbiota in this process is interchangeable and can be played by SRB, methanogenic archaea, or acetogenic bacteria (Vianna et al., 2008). Formation of microbiota with the prevalence of one of these hydrogenotrophic groups may happen by chance. For example, the proportion of periodontal disease cases involving methanogenic archaea at 36% (Lepp et al., 2004) is very similar to the proportion of methane producers in a general population, which remains very stable over a 35-year period despite the extensive use of antibiotics and dietary changes (33.6–36.4%) (Levitt et al., 2006). A similar link may exist between the frequency of SRB in periodontal disease and the general occurrence and prevalence of SRB on other body sites. These hypotheses are worth testing to potentially provide a simple mechanistic explanation for the occurrence and frequency of methanogens and SRBs as hydrogenotrophic microorganisms in periodontitis.

Comparison of metagenomes of healthy and diseased microbiota may help to identify the sets of genes differentially represented in these two conditions and point to the enrichment or reduction of genes specific for pathologies. Signatures of periodontal disease point to the enrichment by genes encoding metabolic functions that are consistent with a parasitic lifestyle and anaerobic metabolism, as well as by genes encoding virulence factors and the biosynthesis of toxic factors (Liu et al., 2012; Wang et al., 2013). The shift of the subgingival crevice microbiota to the anaerobic type in disease may facilitate the colonization of this niche by hydrogenotrophic microorganisms from other body sites in the corresponding carriers.

Another important factor involved in the disease is the host response, which is realized via the host-microbe interface (Armitage, 2013; Bartold and Van Dyke, 2013). More facts are emerging supporting the view that one of the primary causes of the disease is the inappropriate host response to the microbiota leading to tissue changes at the initial gingivitis stage. This altered microenvironment affects the composition of local microbiota, shifting it to the pathobiota, and contributes to the subsequent development of periodontitis, if the genetic and environmental factors are conducive for disease development (Bartold and Van Dyke, 2013). The host mechanisms involved in this destructive process can be switched off in animal models of periodontitis, which result in drastic improvement of clinical presentation. For example, RNAi-mediated silencing of Atp6i prevents bone loss and inflammation in the mouse model of periodontal disease (Jiang et al., 2013) and in the mouse model of endodontic disease (Ma et al., 2013). Silencing of cathepsin K in periapical tissues can significantly reduce endodontic disease development, bone destruction and inflammation in a mouse disease model (Gao et al., 2013). Periodontitis, therefore, is provoked by the excessive host's proinflammatory responses to the microbiota during the early stages of disease development, thus changing the microenvironment and creating novel niche opportunities (Bartold and Van Dyke, 2013), which are promptly used by a number of microorganisms, including the Archaea.

The involvement of cell-mediated immunity in periodontal disease has been first demonstrated more than 40 years ago (Ivanyi et al., 1972). Since then, the idea that the development of periodontal disease with the resulting connective tissue breakdown involves inappropriate host responses has been gaining a widespread acceptance, especially following the discovery of the host modulating properties of minocycline 30 years ago (Golub et al., 1983). This effect is based on inhibition of certain host metal matrix metalloproteinases (MMPs) by low-dose tetracyclines; this type of treatment is approved by the FDA and other national regulatory agencies in Canada and Europe for the management of chronic periodontal disease (Gu et al., 2012). Current preventive and treatment approaches, however, are only partially effective because of the focus on biofilm management (Tonetti et al., 2011). Better understanding of host modulation and inflammation resolution is necessary to develop new, more effective, and efficient preventive and treatment approaches (Tonetti et al., 2011).

But why the disease is so widespread? Have the host responses toward the oral microbiota always been so destructive? These questions can be answered through the prism of dietary and cultural changes in human history. Two major changes in the diet, i.e. the carbohydrate-rich Neolithic with the introduction of farming (~ 10,000 years ago) and the more recent advent of industrially-processed flour and sugar (in ~ 1850), shifted the oral microbial community to a disease-associated configuration (Adler et al., 2013). Thus, the host-microbe equilibrium that had evolved as a result of previous long-term co-evolution has been jeopardized by the diet-affected and less stable and diverse microbiota, with presumably less appropriate functional and signaling properties. This has probably been the key event that continues to incite exaggerated host immune responses leading to the disease.

Currently, there is no substantial evidence supporting the pathogenic properties of the Archaea. The best they can do is to cease the opportunity created by pathological processes and occupy the microenvironments suitable for this type of anaerobic hydrogenotrophic metabolism. These niches, however, can also be occupied by other microbiota with similar metabolic properties.

## References

[B1] Adler C. J. Dobney K. Weyrich L. S. Kaidonis J. Walker A. W. Haak W. (2013). Sequencing ancient calcified dental plaque shows changes in oral microbiota with dietary shifts of the neolithic and industrial revolutions. Nat. Genet. 45, 450–455 10.1038/ng.2536 23416520 PMC3996550

[B2] Albuquerque C. M. Cortinhas A. J. Morinha F. J. Leitão J. C. Viegas C. A. Bastos E. M. (2012). Association of the IL-10 polymorphisms and periodontitis: a meta-analysis. Mol. Biol. Rep. 39, 9319–9329 10.1007/s11033-012-1738-1 22763734

[B3] Armitage G. C. (2013). Learned and unlearned concepts in periodontal diagnostics: a 50-year perspective. Periodontol. 2000, 62, 20–36 10.1111/prd.12006 23574462

[B4] Atanasovska-Stojanovska A. Trajkov D. Popovska M. Spiroski M. (2012). IL10 -1082, IL10 -819 and IL10 -592 polymorphisms are associated with chronic periodontitis in a Macedonian population. Hum. Immunol. 73, 753–758 10.1016/j.humimm.2012.04.009 22537751

[B5] Attaluri A. Jackson M. Valestin J. Rao S. S. (2010). Methanogenic flora is associated with altered colonic transit but not stool characteristics in constipation without IBS. Am. J. Gastroenterol. 105, 1407–1411 10.1038/ajg.2009.655 19953090 PMC3822765

[B6] Bartold P. M. Van Dyke T. E. (2013). Periodontitis: a host-mediated disruption of microbial homeostasis. Unlearning learned concepts. Periodontol. 2000, 62, 203–217 10.1111/j.1600-0757.2012.00450.x 23574467 PMC3692012

[B7a] Belay N. Johnson R. Rajagopal B. S. Conway de Macario E. Daniels L. (1988). Methanogenic bacteria from human dental plaque. Appl. Environ. Microbiol. 54, 600–603 3355146 10.1128/aem.54.2.600-603.1988 PMC202503

[B7] Belay N. Mukhopadhyay B. Conway de Macario E. Galask R. Daniels L. (1990). Methanogenic bacteria in human vaginal samples. J. Clin. Microbiol. 28, 1666–1668 2199527 10.1128/jcm.28.7.1666-1668.1990 PMC268013

[B8] Carbonero F. Benefiel A. C. Alizadeh-Ghamsari A. H. Gaskins H. R. (2012). Microbial pathways in colonic sulfur metabolism and links with health and disease. Front. Physiol. 3:448 10.3389/fphys.2012.00448 23226130 PMC3508456

[B9] Cavicchioli R. Curmi P. M. Saunders N. Thomas T. (2003). Pathogenic archaea: do they exist? Bioessays 25, 1119–1128 10.1002/bies.10354 14579252

[B10] Chatterjee S. Park S. Low K. Kong Y. Pimentel M. (2007). The degree of breath methane production in IBS correlates with the severity of constipation. Am. J. Gastroent. 102, 837–841 10.1111/j.1572-0241.2007.01072.x 17397408

[B11] Chow J. Tang H. Mazmanian K. S. (2011). Pathobionts of the gastrointestinal microbiota and inflammatory disease. Curr. Opin. Immunol. 23, 473–480 10.1016/j.coi.2011.07.010 21856139 PMC3426444

[B12] Di Stefano M. Corazza G. R. (2010). Methanogenic flora and constipation: many doubts for a pathogenetic link. Am. J. Gastroenterol. 105, 2304–2305 10.1038/ajg.2010.282 20927078

[B13] Divaris K. Monda K. L. North K. E. Olshan A. F. Lange E. M. Moss K. (2012). Genome-wide association study of periodontal pathogen colonization. J. Dent. Res. 91 Suppl. 7, 21S–28S 10.1177/0022034512447951 22699663 PMC3383103

[B14] Dridi B. Henry M. El Khéchine A. Raoult D. Drancourt M. (2009). High prevalence of *Methanobrevibacter smithii* and *Methanosphaera stadtmanae* detected in the human gut using an improved DNA detection protocol. PLoS ONE 4:e7063 10.1371/journal.pone.0007063 19759898 PMC2738942

[B15] Eckburg P. B. Lepp P. W. Relman D. A. (2003). Archaea and their potential role in human disease. Infect. Immun. 71, 591–596 10.1128/IAI.71.2.591-596.2003 12540534 PMC145348

[B15a] Ferrari A. Brusa T. Rutili A. Canzi E. Biavati B. (1994). Isolation and characterization Methanobrevibacter oralis sp. nov. Curr Microbiol. 6, 7–12

[B16] Fiedorek S. C. Pumphrey C. L. Casteel H. B. (1990). Breath methane production in children with constipation and encopresis. J. Pediatr. Gastroenterol. Nutr. 10, 473–477 10.1097/00005176-199005000-00010 2162940

[B17] Florin T. H. Zhu G. Kirk K. M. Martin N. G. (2000). Shared and unique environmental factors determine the ecology of methanogens in humans and rats. Am. J. Gastroenterol. 95, 2872–2879 10.1111/j.1572-0241.2000.02319.x 11051362

[B18] Furnari M. Savarino E. Bruzzone L. Moscatelli A. Gemignani L. Giannini E. G. (2012). Reassessment of the role of methane production between irritable bowel syndrome and functional constipation. J. Gastrointestin. Liver Dis. 21, 157–163 22720304

[B19] Gao B. Chen W. Hao L. Zhu G. Feng S. Ci H. (2013). Inhibiting periapical lesions through AAV-RNAi silencing of cathepsin K. J. Dent. Res. 92, 180–186 10.1177/0022034512468757 23166044 PMC3545691

[B20] Golub L. M. Lee H. M. Lehrer G. Nemiroff A. McNamara T. F. Kaplan R. (1983). Minocycline reduces gingival collagenolytic activity during diabetes. Preliminary observations and a proposed new mechanism of action. J. Periodontal. Res. 18, 516–526 10.1111/j.1600-0765.1983.tb00388.x 6315909

[B21] Gu Y. Walker C. Ryan M. E. Payne J. B. Golub L. M. (2012). Non-antibacterial tetracycline formulations: clinical applications in dentistry and medicine. J. Oral. Microbiol. 4 10.3402/jom.v4i0.19227 23071896 PMC3471324

[B22] Hajishengallis G. Darveau R. P. Curtis M. A. (2012). The keystone-pathogen hypothesis. Nat. Rev. Microbiol. 10, 717–725 10.1038/nrmicro2873 22941505 PMC3498498

[B23] Heggendorn F. L. Souza Gonçalves L. Dias E. P. Silva Junior A. Galvão M. M. Lutterbach M. T. (2013). Detection of sulphate-reducing bacteria in human saliva. Acta Odontol. Scand. [Epub ahead of print]. 10.3109/00016357.2013.770163 23638810

[B24] Ivanyi L. M. Wilton J. M. A. Lehner T. (1972). Cell-mediated immunity in periodontal disease; cytotoxicity, migration inhibition and lymphocyte transformation studies. Immunology 22, 141–145 5013909 PMC1408213

[B25] Jiang H. Chen W. Zhu G. Zhang L. Tucker B. Hao L. (2013). RNAi-mediated silencing of Atp6i and Atp6i haploinsufficiency prevents both bone loss and inflammation in a mouse model of periodontal disease. PLoS ONE 8:e58599 10.1371/journal.pone.0058599 23577057 PMC3618217

[B26] Khachatryan Z. A. Ktsoyan Z. A. Manukyan G. P. Kelly D. Ghazaryan K. A. Aminov R. I. (2008). Predominant role of host genetics in controlling the composition of gut microbiota. PLoS ONE 3:e3064 10.1371/journal.pone.0003064 18725973 PMC2516932

[B27] Khelaifia S. Raoult D. Drancourt M. (2013). A versatile medium for cultivating methanogenic archaea. PLoS ONE 8:e61563 10.1371/journal.pone.0061563 23613876 PMC3629087

[B28] Lepp P. W. Brinig M. M. Ouverney C. C. Palm K. Armitage G. C. Relman D. A. (2004). Methanogenic Archaea and human periodontal disease. Proc. Natl. Acad. Sci. U.S.A. 101, 6176–6181 10.1073/pnas.0308766101 15067114 PMC395942

[B29] Levitt M. D. Furne J. K. Kuskowski M. Ruddy J. (2006). Stability of human methanogenic flora over 35 years and a review of insights obtained from breath methane measurements. Clin. Gastroenterol. Hepatol. 4, 123–129 10.1016/j.cgh.2005.11.006 16469670

[B30] Lewis S. Cochrane S. (2007). Alteration of sulfate and hydrogen metabolism in the human colon by changing intestinal transit rate. Am. J. Gastroenterol. 102, 624–633 10.1111/j.1572-0241.2006.01020.x 17156141

[B31] Littman D. R. Pamer E. G. (2011). Role of the commensal microbiota in normal and pathogenic host immune responses. Cell Host Microbe 10, 311–323 10.1016/j.chom.2011.10.004 22018232 PMC3202012

[B32] Liu B. Faller L. L. Klitgord N. Mazumdar V. Ghodsi M. Sommer D. D. (2012). Deep sequencing of the oral microbiome reveals signatures of periodontal disease. PLoS ONE 7:e37919 10.1371/journal.pone.0037919 22675498 PMC3366996

[B33] Ma J. Chen W. Zhang L. Tucker B. Zhu G. Sasaki H. (2013). RNA interference-mediated silencing of Atp6i prevents both periapical bone erosion and inflammation in the mouse model of endodontic disease. Infect. Immun. 81, 1021–1030 10.1128/IAI.00756-12 23166162 PMC3639609

[B34] McKay L. F. Eastwood M. A. Brydon W. G. (1985). Methane excretion in man – a study of breath, flatus, and faeces. Gut 26, 69–74 10.1136/gut.26.1.69 3965369 PMC1432392

[B35] Medani M. Collins D. Docherty N. G. Baird A. W. O'Connell P. R. Winter D. C. (2011). Emerging role of hydrogen sulfide in colonic physiology and pathophysiology. Inflamm. Bowel Dis. 17, 1620–1625 10.1002/ibd.21528 21674719

[B36] Miller T. L. Wolin M. J. (1982). Enumeration of Methanobrevibacter smithii in human feces. Arch. Microbiol. 131, 14–18 10.1007/BF00451492 7065811

[B37] Miller T. L. Wolin M. J. (1985). *Methanosphaera stadtmaniae* gen. nov., sp. nov.: a species that forms methane by reducing methanol with hydrogen. Arch. Microbiol. 141, 116–122 10.1007/BF00423270 3994486

[B38] Peters B. M. Jabra-Rizk M. A. O'May G. A. Costerton J. W. Shirtliff M. E. (2012). Polymicrobial interactions: impact on pathogenesis and human disease. Clin. Microbiol. Rev. 25, 193–213 10.1128/CMR.00013-11 22232376 PMC3255964

[B39] Pimentel M. Mayer A. G. Park S. Chow E. J. Hasan A. Kong Y. (2003). Methane production during lactulose breath test is associated with gastrointestinal disease presentation. Dig. Dis. Sci. 48, 86–92 10.1023/A:1021738515885 12645795

[B39a] Scanlan P. D. Shanahan F. Marchesi J. R. (2008). Human methanogen diversity and incidence in healthy and diseased colonic groups using mcrA gene analysis. BMC Microbiol. 8:79 10.1186/1471-2180-8-79 18492229 PMC2408590

[B40] Socransky S. S. Haffajee A. D. Smith C. Martin L. Haffajee J. A. Uzel N. G. (2004). Use of checkerboard DNA-DNA hybridization to study complex microbial ecosystems. Oral. Microbiol. Immunol. 19, 352–362 10.1111/j.1399-302x.2004.00168.x 15491460

[B41] Stringer A. M. Al-Dasooqi N. Bowen J. M. Tan T. H. Radzuan M. Logan R. M. (2013). Biomarkers of chemotherapy-induced diarrhea: a clinical study of intestinal microbiome alterations, inflammation and circulating matrix metalloproteinases. Support. Care Cancer 21, 1843–1852 10.1007/s00520-013-1741-7 23397098

[B42] Tonetti M. S. Chapple I. L. Working Group 3 of Seventh European Workshop on Periodontology (2011). Biological approaches to the development of novel periodontal therapies–consensus of the Seventh European Workshop on Periodontology. J. Clin. Periodontol. 38 Suppl. 11, 114–118 10.1111/j.1600-051X.2010.01675.x 21323708

[B43] Vianna M. E. Conrads G. Gomes B. P. F. A. Horz H. P. (2006). Identification and quantification of archaea involved in primary endodontic infections. J. Clin. Microbiol. 44, 1274–1282 10.1128/JCM.44.4.1274-1282.2006 16597851 PMC1448633

[B44] Vianna M. E. Holtgraewe S. Seyfarth I. Conrads G. Horz H. P. (2008). Quantitative analysis of three hydrogenotrophic microbial groups, methanogenic archaea, sulfate-reducing bacteria, and acetogenic bacteria, within plaque biofilms associated with human periodontal disease. J. Bacteriol. 190, 3779–3785 10.1128/JB.01861-07 18326571 PMC2394984

[B45] Wang J. Qi J. Zhao H. He S. Zhang Y. Wei S. (2013). Metagenomic sequencing reveals microbiota and its functional potential associated with periodontal disease. Sci. Rep. 3, 1843 10.1038/srep01843 23673380 PMC3654486

[B46] Weaver G. A. Krause J. A. Miller T. L. Wolin M. J. (1986). Incidence of methanogenic bacteria in a sigmoidoscopy population, an association of methanogenic bacteria and diverticulosis. Gut 27, 698–704 10.1136/gut.27.6.698 3721294 PMC1433329

[B47] Zhong Q. Ding C. Wang M. Sun Y. Xu Y. (2012). Interleukin-10 gene polymorphisms and chronic/aggressive periodontitis susceptibility: a meta-analysis based on 14 case-control studies. Cytokine 60, 47–54 10.1016/j.cyto.2012.05.014 22698805

